# Charting a sustainable future in radiology: evaluating radiologists’ knowledge, attitudes, and practices toward environmental responsibility

**DOI:** 10.1186/s13244-025-01917-7

**Published:** 2025-02-17

**Authors:** Mohamed M. Abuzaid, Nora Almuqbil

**Affiliations:** 1 Medical Diagnostic Imaging Department, College of Health Sciences, University of Sharjah, Sharjah, UAE; 2 Department of Radiological Sciences, College of Health and Rehabilitation Sciences, Princess Nourah Bint Abdulrahman University, P.O. Box 84428, Riyadh, 11671 Saudi Arabia

**Keywords:** Radiology, Sustainability, Environmental responsibility, Green healthcare, Radiologist attitudes

## Abstract

**Objectives:**

This study assesses radiologists’ knowledge, attitudes, and practices regarding healthcare sustainability. With radiology’s substantial environmental impact, sustainable practices are crucial to reduce energy use, waste, and resource depletion. The study evaluates radiologists’ awareness, engagement, and perceived barriers to sustainable practices in the UAE, identifying areas for improvement and intervention.

**Methods:**

A cross-sectional survey was conducted among UAE radiologists in hospitals, clinics, and medical centers from August to October 2024. Developed and piloted by the research team, the survey addressed demographic details, sustainability knowledge, attitudes, current practices, and implementation barriers. Convenience sampling yielded 111 responses, analyzed using descriptive and inferential statistics to identify trends and associations.

**Results:**

The findings indicate moderate knowledge levels, with 31.8% of radiologists agreeing to understand sustainability concepts. While 36.4% strongly agreed on its importance, only 20% felt it was prioritized at their workplace. Key barriers included lack of training (40.5% agreed, 29.7% strongly agreed) and insufficient financial support (37.8% agreed, 25.2% strongly agreed). Digital documentation and waste-reduction practices were observed but varied in consistency.

**Conclusion:**

Radiologists display a positive attitude toward sustainability but face significant implementation barriers, primarily in institutional support and resources. Addressing training gaps and increasing leadership commitment are essential to advancing sustainable practices. Future initiatives should emphasize policy support, education, and resource allocation to foster a sustainable radiology sector.

**Critical relevance statement:**

This article critically examines radiologists’ knowledge, attitudes, and barriers to sustainable practices, highlighting the need for institutional support and targeted training to advance environmental responsibility and sustainable practices within clinical radiology.

**Key Points:**

- Radiologists support sustainability but lack knowledge of specific practices.
- Key challenges include limited training, support, and funding.
- Commitment, training, and resources are essential for sustainable radiology.

**Graphical Abstract:**

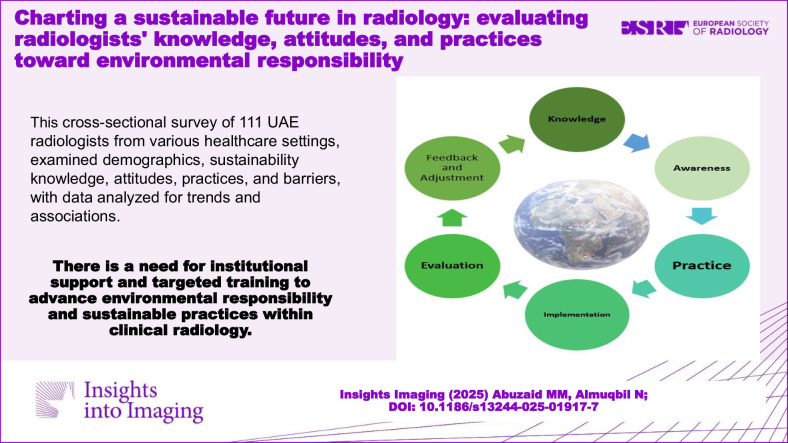

## Introduction

The healthcare sector, particularly radiology, has a significant environmental footprint due to the high energy demands, resource consumption, and waste of imaging equipment and practices. Growing awareness of climate change and its adverse health effects has sparked a global shift towards sustainable practices across medical fields, including radiology [1, 2] Since the healthcare industry contributes approximately 8% of all greenhouse gas emissions globally, radiology’s role in environmental sustainability is becoming increasingly relevant [3].

Radiology departments are responsible for delivering high-quality patient care while minimizing their environmental impact. Traditional imaging methods, such as CT and MRI, consume substantial energy, with a single MRI scanner consuming up to 525,600 kWh annually [4]. Initiatives to reduce resource use in radiology include optimizing imaging protocols, transitioning to cloud-based picture archiving and communication systems (PACS), and adopting low-energy lighting and real-time power monitoring tools [5]. Moreover, digital documentation, waste recycling, and environmentally conscious procurement have demonstrated substantial energy and cost savings in radiology [6]. Sustainability in radiology, often called “Green Radiology,” includes integrating environmental, economic, and social dimensions into medical imaging. The Canadian Association of Radiologists emphasizes the need for radiology to adopt environmentally sustainable practices to counter climate-related health risks, such as extreme weather events that disrupt healthcare delivery [7]. This paradigm shift toward green radiology is expected to support environmental preservation and economic efficiency. Despite the recognized importance of sustainability, there remains a gap in radiologists’ awareness and adoption of environmentally sustainable practices. Many radiologists report limited knowledge about sustainable options and often lack the resources or support to implement greener practices effectively [8]. Barriers such as Inadequate leadership support, lack of funding, and time constraints are frequently cited as obstacles to advancing sustainability in radiology departments. Addressing these challenges requires a systematic approach, encompassing education, leadership commitment, and policy development to foster sustainable radiology practices [9]. This study aims to investigate radiologists’ knowledge, attitudes, and practices concerning environmental sustainability within the radiology department.

The study seeks to provide actionable insights to support the transition toward green radiology by identifying current practices and perceived barriers to sustainability. Understanding radiologists’ perspectives on sustainability will enable the development of targeted interventions, potentially positioning radiology at the forefront of sustainable healthcare.

## Methods

### Study design

This cross-sectional study used a quantitative approach to gather data from practising radiologists in the United Arab Emirates (UAE) through a structured questionnaire. The research team crafted the survey utilized in this study to evaluate the current understanding, attitudes, and practices regarding sustainability among radiologists. The survey underwent a comprehensive development process, including initial design, pilot testing, and subsequent refinement, to ensure the questions’ clarity, relevance, and reliability. Feedback from the pilot study was used to modify, optimize, and ensure that the survey captured relevant insights effectively.

### Survey structure

The survey comprised multiple sections, each tailored to capture specific dimensions of the respondents’ experiences, knowledge, and perspectives on sustainability in healthcare.

Section one collected the participant’s demographic information, which included gender, age, years of experience in radiology, and type of healthcare facility (e.g., hospital, clinic, research institution). Section two investigated the participants’ knowledge of sustainability in terms of the definition of sustainability and key areas of focus for sustainable healthcare, such as waste management, energy efficiency, and water conservation. Familiarity with specific sustainability initiatives at their workplace. Section three investigates radiologists’ attitudes toward sustainability. It includes statements where participants rate their level of agreement, covering topics such as: “Importance of sustainability in healthcare”, “Perceived benefits of implementing sustainable practices for patient outcomes”, and “Support for integrating more sustainable practices into their professional routines” Section four investigate the radiologist’s sustainability Practices. This section examined the frequency and types of sustainable practices radiologists currently implement in their daily activities. Participants are asked to indicate the following practices they engage in regularly: energy conservation, waste reduction, and use of environmentally friendly products. Frequency of participation in sustainability initiatives at their workplace. Section five examined the barriers and facilitators in identifying perceived obstacles and enablers that influence adopting sustainability practices in radiology departments. Section six examined behavioral Intent and Motivation, including motivation to learn more about sustainability, encouragement of others to adopt sustainable practices and suggestions for improving sustainability within their departments. Section seven examined the impact and Evaluation of Sustainability Initiatives using Likert-scale statements to assess perceptions of sustainability’s impact on healthcare. Statements include awareness of sustainability’s impact on patient care and environmental outcomes, regularity of updating knowledge on sustainability, and engagement in evaluating the effectiveness of sustainability practices. It also included statements about organizational support and communication regarding sustainability benefits, highlighting the role of leadership in fostering a sustainable culture.

The last section examined barriers to implementing sustainability in the radiology department, including lack of training, financial support, workload, and staffing shortages in prioritizing sustainability and leadership commitment to sustainability initiatives.

### Participants and data collection procedures

The study targeted radiologists in various healthcare settings, including hospitals, clinics, and diagnostic centers in the UAE. Eligibility criteria included current employment as a radiologist in a healthcare institution and willingness to participate in a sustainability-related survey. The study used convenience sampling to collect data from radiologists between August and October 2024. Convenience sampling was chosen due to the practical limitations of identifying the total population of radiologists in the country, allowing the researchers to gather responses from a readily accessible subset of radiologists. During the three months, survey invitations were distributed across healthcare institutions, professional networks, and relevant forums, reaching willing and available radiologists to participate. This approach, while not guaranteeing a fully representative sample, enabled the collection of valuable insights into the knowledge, attitudes, and practices of radiologists concerning sustainability within a defined timeframe. The convenience sampling method facilitated efficient data collection, yielding 110 completed surveys that provide a snapshot of current perspectives on sustainability in radiology.

### Data analysis

Data analysis involved descriptive and inferential statistics to evaluate radiologists’ knowledge, attitudes, practices, and perceived barriers to sustainability. Mann–Whitney *U*-tests assessed gender-based differences, while Kruskal–Wallis *H*-tests explored variations by facility and energy-saving engagement. Significant differences were noted in knowledge, attitudes, and practices based on sustainability engagement frequency, with analyses conducted in SPSS at a significance level of *p* < 0.05.

### Ethical approval

The Research Ethics Committee of the University of Sharjah approved this study (REC-24-05-27-01-F). All participants provide informed consent, and participation is voluntary, with the option to withdraw immediately. Confidentiality was assured through anonymous data collection; no personally identifiable information was recorded. All data was securely stored and accessible only to the principal investigator and designated research team members in compliance with ethical guidelines on participant privacy and data protection.

## Results

### Demographic characteristics of surveyed radiologists

**Fig. 1:**
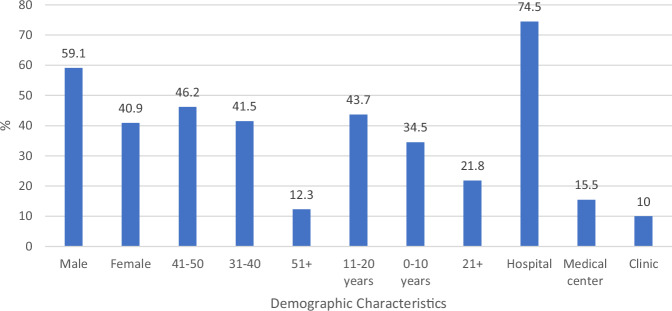
Demographic characteristics of surveyed radiologists

With an estimated response rate of 84%, approximately 131 radiologists were initially approached. Figure 1 summarizes the demographic characteristics of the 110 radiologists who completed the survey. Most participants were male (59.1%), with the largest age group being 41–50 (46.2%). In terms of experience, 44.9% of respondents had 11–20 years of experience in radiology. Most participants worked in hospitals (74.5%), while a smaller percentage were employed at medical centers (15.5%) and clinics (10.0%).

### Focus and implementation of sustainability practices among radiologists

Table S1 presents an overview of radiologists’ understanding, prioritization, and implementation of sustainability practices. Over half (50.9%) of the respondents view sustainability in healthcare as primarily about reducing environmental impact. When asked about key areas of focus in sustainable healthcare, 72.7% indicated all key areas—waste management, energy efficiency, and water conservation—as important. In clinical decision-making, 39.1% regard sustainability as a primary consideration, while 41.8% see it as secondary to patient care.

The biggest barriers to implementing sustainable practices were cited as lack of awareness and training (50.0%) and insufficient managerial support (26.4%). Regarding waste disposal methods, 42.7% are familiar with recycling, while 40.9% are acquainted with incineration. Regarding energy reduction practices, 34.5% engage rarely, with only 18.2% engaging daily. Common sustainable practices incorporated into daily routines include digital documentation (54.5%) and the use of reusable materials (22.7%), though fewer respondents use energy-efficient lighting (14.5%) or water-saving fixtures (8.2%).

### Radiologists’ knowledge and awareness of sustainability concepts

Table 1 outlines radiologists’ self-reported knowledge and awareness regarding various sustainability concepts in healthcare. A majority (31.8%) agreed that they understand the concept of sustainability, with an additional 25.5% strongly agreeing. However, familiarity with workplace sustainability policies was lower, with only 14.6% agreeing and 8.2% strongly agreeing, while 25.5% disagreed and 17.3% strongly disagreed.

**Table 1:** Radiologists’ knowledge and awareness of sustainability concepts

	Strongly disagree	Disagree	Neutral	Agree	Strongly agree
	*n* (%)				
Understand sustainability concept	11 (10.0)	10 (9.1)	26 (23.6)	35 (31.8)	28 (25.5)
Aware of sustainability impact on patient care	9 (8.2)	14 (12.7)	39 (35.5)	35 (31.8)	13 (11.8)
Familiar with workplace sustainability policies	19 (17.3)	28 (25.5)	38 (34.6)	16 (14.6)	9 (8.2)
Identify benefits of sustainable practices in healthcare	11 (10.0)	14 (12.7)	32 (29.1)	28 (25.5)	25 (22.7)
Regularly update knowledge of sustainability	13 (11.8)	24 (21.8)	27 (24.6)	29 (26.4)	17 (15.5)
Aware of the environmental impact of healthcare waste	12 (10.9)	13 (11.8)	33 (30.0)	25 (22.7)	27 (24.6)
Understand energy conservation in a healthcare setting	11 (10.0)	12 (10.9)	29 (26.4)	28 (25.5)	30 (27.3)

When asked about the impact of sustainability on patient care, 31.8% agreed that they were aware, although 35.5% remained neutral. Many respondents also acknowledged the environmental impact of healthcare waste (22.7% agree, 24.6% strongly agree) and understood the importance of energy conservation within healthcare settings (25.5% agree, 27.3% strongly agree). Overall, the responses indicate varying levels of knowledge and awareness, with some areas, such as workplace policy familiarity, showing potential for improvement in sustainability training and awareness initiatives.

### Radiologists’ attitudes towards sustainability in healthcare

Table 2 presents data on radiologists’ attitudes towards sustainability within the healthcare sector. A substantial majority (36.4%) strongly agreed that sustainability is important in healthcare, with 23.6% agreeing. Similarly, 31.8% strongly agreed that sustainable practices are essential for improving patient outcomes, while 18.2% agreed. However, only 20.0% strongly agreed that sustainability is prioritized at their workplace, and 21.8% disagreed, indicating potential gaps in institutional commitment.

**Table 2:** Radiologists’ attitudes towards sustainability in healthcare

	Strongly disagree	Disagree	Neutral	Agree	Strongly agree
	*n* (%)				
Sustainability is important in healthcare	7 (6.4)	10 (9.1)	27 (24.5)	26 (23.6)	40 (36.4)
Sustainable practices are essential for improving patient outcomes.	13 (11.8)	10 (9.1)	32 (29.1)	20 (18.2)	35 (31.8)
Sustainability is prioritized in the workplace.	10 (9.1)	24 (21.8)	40 (36.4)	14 (12.7)	22 (20.0)
Integrate sustainability into all health professional training programs.	8 (7.3)	13 (11.8)	24 (21.8)	29 (26.4)	36 (32.7)
Motivated to learn more about sustainable healthcare practices.	7 (6.4)	8 (7.3)	32 (29.1)	40 (36.4)	23 (20.9)
Every health professional can promote sustainability.	8 (7.3)	6 (5.5)	32 (29.1)	26 (23.6)	38 (34.5)
The healthcare sector can reduce its environmental impact.	11 (10.0)	11 (10.0)	34 (30.9)	29 (26.4)	25 (22.7)

The integration of sustainability into all health professional training programs was strongly supported, with 32.7% strongly agreeing and 26.4% agreeing. When asked about personal motivation to learn more about sustainable practices, 20.9% strongly agreed, though a large portion (29.1%) remained neutral. Additionally, 34.5% strongly agreed that every health professional can promote sustainability, and 26.4% agreed that the healthcare sector has the potential to reduce its environmental impact. These findings suggest a positive attitude toward sustainability among radiologists, though there is a perceived need for greater institutional support and prioritization.

### Radiologists’ practices related to sustainability in healthcare

Table 3 summarizes radiologists’ reported practices concerning sustainability. A majority (33.3%) agreed that they follow sustainability guidelines, although only 3.6% strongly agreed, indicating moderate adherence to established protocols. Regarding waste reduction, responses were mixed, with 26.1% disagreeing and only 6.3% strongly agreeing, suggesting that waste management practices may need to be consistently prioritized.

**Table 3:** Radiologists’ practices related to sustainability in healthcare

	Strongly disagree	Disagree	Neutral	Agree	Strongly agree
	*n* (%)				
Follow guidelines	17 (15.3)	18 (16.2)	34 (30.6)	37 (33.3)	4 (3.6)
Reduce waste	22 (19.8)	29 (26.1)	27 (24.3)	25 (22.5)	7 (6.3)
Use resources responsibly	13 (11.7)	11 (9.9)	32 (28.8)	31 (27.9)	23 (20.7)
Consider the impact of decisions	14 (12.6)	22 (19.8)	39 (35.1)	29 (26.1)	6 (5.4)
Support energy initiatives	9 (8.1)	18 (16.2)	27 (24.3)	38 (34.2)	18 (16.2)
Follow disposal procedures	17 (15.3)	25 (22.5)	32 (28.8)	14 (12.6)	22 (19.8)
Seek product alternatives	21 (18.9)	32 (28.8)	31 (27.9)	15 (13.5)	11 (9.9)

Regarding resource use, 27.9% agreed, and 20.7% strongly agreed that they use resources responsibly. Similarly, while 34.2% agreed with supporting energy-saving initiatives, a lower percentage (16.2%) strongly agreed. Disposal procedure adherence varied, with 28.8% remaining neutral and 19.8% strongly agreeing. Regarding considering environmental impact in decision-making, 26.1% agreed, though 19.8% disagreed. Only a minority actively seek sustainable product alternatives, with 28.8% disagreeing and 18.9% strongly disagreeing. These results highlight moderate engagement in sustainable practices, with room for improvement, particularly in waste reduction and product selection.

### Perceived barriers to implementing sustainability in radiology

Table S2 outlines the perceived barriers that radiologists face in implementing sustainable practices. A significant proportion of respondents (40.5%) agreed that a lack of training hinders sustainability efforts, with an additional 29.7% strongly agreeing. Similarly, financial support was cited as a barrier, with 37.8% agreeing and 25.2% strongly agreeing that insufficient funding impacts their ability to adopt sustainable practices.

Workload and staffing shortages were also identified as obstacles, with 29.7% agreeing and 25.2% strongly agreeing that these factors limit their capacity to prioritize sustainability. Many respondents (36.9% agree, 27.9% strongly agree) indicated that a lack of leadership commitment impedes sustainable initiatives. Finally, while many (37.8%) agreed that better communication of sustainability benefits is needed, only 15.3% strongly agreed, suggesting that communication is a concern but may be less pressing than other barriers. These results highlight training, financial support, and leadership commitment as key areas requiring improvement to facilitate sustainable practices in radiology.

### Analysis of radiologists’ knowledge, attitudes, practices, and perceived barriers to sustainability

The analysis of participant scores across categories reveals that higher scores signify stronger agreement in sustainability knowledge, positive attitudes, and sustainable practices, while higher barrier scores indicate perceived challenges.

In the Knowledge category, scores ranged from 6.4%, scoring the lowest, to 7.3%, scoring the highest. Attitude scores ranged from 5.5% at the lowest to 4.5% at the highest. Practice scores showed a wider disparity, with 6.4% scoring the lowest and only 0.9% achieving the highest. For Barriers, 5.5% scored lowest and 1.8% scored highest.

Mann–Whitney *U*-tests revealed that male participants scored significantly higher in knowledge (*z* = −2.073, *p* = 0.038) and practice (*z* = −2.775, *p* = 0.006) than females. However, no gender differences were found in Attitude or Barriers. The Kruskal–Wallis *H*-test found no significant differences across facilities in any category. Still, differences were significant in Knowledge (*H* = 16.805, *p* = 0.002), Attitude (*H* = 18.779, *p* < 0.001), and Practice (*H* = 25.747, *p* < 0.001) based on the frequency of energy-saving practices, with monthly and daily participants scoring highest. Barrier scores showed no significant differences based on engagement frequency.

## Discussion

The results of this study reveal important insights into the current state of sustainability practices among radiologists in the United Arab Emirates, highlighting both progress and ongoing challenges. A significant proportion of respondents (50.9%) view sustainability primarily as reducing environmental impact, echoing the sentiments expressed in previous studies by Rawashdeh et al [8] and Ghotra et al [10], which similarly emphasize awareness of sustainability’s relevance in healthcare. However, despite this awareness, a notable gap needs to be addressed in adopting specific sustainable practices, with only 14.5% of participants regularly using energy-efficient lighting. This discrepancy aligns with Sumner et al [4] findings, which identified institutional barriers, particularly lack of managerial support and training, as critical impediments to implementing effective sustainability initiatives [4]. As such, while radiologists desire to engage in sustainable practices, integrating these practices into daily operations requires targeted interventions that address the identified barriers and enhance institutional commitment to sustainability.

The findings align with previous literature and underscore environmental sustainability’s gradual but critical integration into radiological practice. Radiologists displayed varying levels of awareness and knowledge about sustainability, with approximately 31.8% indicating a sound understanding. However, the knowledge gap emphasizes the need for enhanced educational initiatives focusing on the environmental impact of radiology practices.

Our study highlights discrepancies in sustainable practices, with over half of the respondents adopting digital documentation. Meanwhile, more energy-intensive measures, such as implementing energy-efficient lighting, must catch up at 14.5% adoption. A similar trend was noted by another study, where lower-cost, accessible sustainability measures were more readily adopted than those requiring higher upfront investments or operational changes [1]. This indicates a pattern where easily accessible and low-cost practices are preferred, yet broader systemic changes face resistance, often due to perceived financial and operational burdens.

Radiologists in this study acknowledged the importance of sustainability, with 36.4% agreeing that it is essential in healthcare. This attitude aligns with sentiments documented by Currie et al [11], where a similar proportion of radiologists supported sustainable practices to improve patient care outcomes. Nevertheless, the disparity in perceived prioritization at the workplace reflects the need for institutional alignment with these values to foster a more comprehensive adoption of sustainability in radiology.

Institutional barriers, such as limited managerial support, emerged as prominent obstacles, with 26.4% of participants citing this as a major deterrent to sustainable practice. This reflects the findings of Bwanga et al [12], who identified a lack of leadership commitment as a critical impediment to sustainability efforts in healthcare. Both studies suggest that sustainability initiatives struggle to gain traction without robust institutional support despite the evident interest among radiologists. To address managerial support gaps, forming sustainability committees within radiology departments can foster accountability and leadership involvement. Incentivizing measurable goals, integrating sustainability metrics into performance reviews, and highlighting cost-saving benefits can align leadership priorities with sustainability. Leadership training on implementing sustainable practices further ensures effective engagement and commitment.

Finally, financial constraints were another significant barrier, with 37.8% of participants agreeing that funding limitations impact adopting sustainable practices. This concurs with Sumner et al [4], who emphasized the role of economic barriers in sustainability efforts within radiology departments [13]. Such financial challenges highlight the need for policies and resources specifically allocated to sustainable initiatives within radiology, suggesting that long-term investments in sustainability can yield both environmental and economic benefits.

In conclusion, this study affirms that while radiologists are increasingly aware and supportive of sustainability in healthcare, significant institutional, educational, and financial barriers hinder the full implementation of green practices. Future strategies should address these barriers by fostering institutional commitment, promoting financial investment, and prioritizing sustainability education tailored to radiology practices.

## Recommendations and targeted strategies for promoting sustainability in radiology

**Fig. 2:**
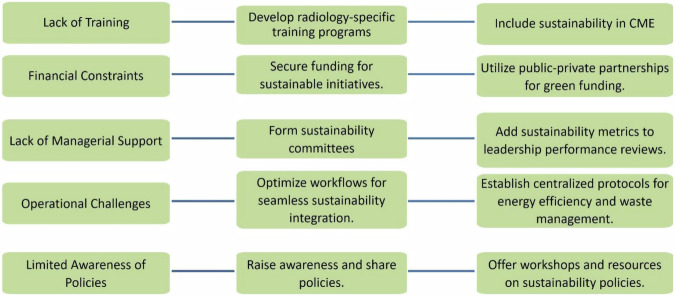
Overcoming barriers to sustainability in radiology: strategies and solutions

A multifaceted approach is essential to advancing sustainability within radiology, as illustrated in Fig. 2, “Overcoming Barriers to Sustainability in Radiology: Strategies and Solutions,” highlighting key barriers and actionable strategies to address them. Sustainability education should be enhanced through training programs focusing on imaging practices’ environmental impact and practical strategies for sustainable radiology. Institutional support must be strengthened by encouraging leadership to prioritize sustainability, allocate dedicated funding, and establish clear policies for environmental responsibility. Forming sustainability committees within radiology departments can foster accountability, and providing incentives for measurable sustainability achievements can further drive institutional commitment. Additionally, targeted initiatives should be implemented, such as introducing energy-efficient technologies and waste-reduction practices. Policy and funding are critical, requiring advocacy for governmental and institutional policies that allocate resources for green technologies and waste management. Public-private partnerships could further enhance the availability of such resources.

Operational changes should include centralized energy-saving protocols and prioritizing the procurement of eco-friendly equipment to streamline sustainable practice adoption without adding significant burdens to radiologists. Finally, ongoing research is necessary to identify the most effective strategies tailored to radiology departments’ unique needs. Together, these recommendations and targeted strategies provide a comprehensive framework to overcome barriers and promote a more environmentally responsible radiology sector.

## Supplementary information

ELECTRONIC SUPPLEMENTARY MATERIAL

## Data Availability

Data will be available upon request from the corresponding author.

## Abbreviation

MRI: Magnetic resonance imaging
