# Supplementary material for: Charting a sustainable future in radiology: evaluating radiologists’ knowledge, attitudes, and practices toward environmental responsibility
Source: Insights Imaging. 2025 Feb 17;16:39. doi: 10.1186/s13244-025-01917-7 (PMC11832974; doi:10.1186/s13244-025-01917-7)
Supplement: Supplementary file 1 — ELECTRONIC SUPPLEMENTARY MATERIAL [file 13244_2025_1917_MOESM1_ESM.pdf]

Charting a Sustainable Future in Radiology: Evaluating Radiologists' Knowledge, Attitudes, and Practices Toward Environmental Responsibility

ELECTRONIC SUPPLEMENTARY MATERIAL

Table S1: Focus and Implementation of Sustainability Practices among Radiologists

		n (%)
Which statement best describes sustainability?	Reducing the environmental impact	56 (50.9)
	Increasing the financial profits	24 (21.8)
	Improve patient comfort regardless of cost	13 (11.8)
	None of the above	17 (15.5)
What are the key areas of focus in sustainability?	Waste Management	15 (13.6)
	Energy efficiency	11 (10.0)
	Water conservation	4 (3.6)
	All the above	80 (72.7)
How do you prioritise sustainability in your clinical decisions?	I do not consider it at all	13 (11.8)
	I consider it when it's convenient	8 (7.3)
	It's of some importance	46 (41.8)
	It's a primary consideration	43 (39.1)
What is the biggest barrier to implementing sustainable practices?	Lack of awareness and training	55 (50.0)
	Insufficient Managerial support	29 (26.4)
	Financial constraints	20 (18.2)
	Perceived impact on patient care	6 (5.5)
What waste disposal methods are you familiar with?	Recycling	47 (42.7)
	Incineration	45 (40.9)
	Chemical treatment	14 (12.7)
	Composting	4 (3.6)
How often do you use practices to reduce energy consumption?	Never	21 (19.1)
	Rarely	38 (34.5)
	Daily	20 (18.2)
	Weekly	10 (9.1)
	Monthly	21 (19.1)
Which sustainable practices are incorporated into your daily routine? (Select all that apply)	Digital documentation	60 (54.5)
	Using reusable materials	25 (22.7)
	Energy-efficient lighting	16 (14.5)
	Water-saving fixtures	9 (8.2)

Table S2: Perceived Barriers to Implementing Sustainability in Radiology

		Strongly Disagree	Disagree	Neutral	Agree	Strongly Agree
		n (%)				
Barriers	Lack of training	8 (7.2)	6 (5.4)	18 (16.2)	45 (40.5)	33 (29.7)
	Financial support	8 (7.2)	9 (8.1)	23 (20.7)	42 (37.8)	28 (25.2)
	Workload and staff shortages	7 (6.3)	14 (12.6)	28 (25.2)	33 (29.7)	28 (25.2)
	Leadership commitment	8 (7.2)	4 (3.6)	26 (23.4)	41 (36.9)	31 (27.9)
	Benefits communication	9 (8.1)	5 (4.5)	37 (33.3)	42 (37.8)	17 (15.3)
